# Effect of early activation and perimodiolar electrodes on cochlear implant impedance

**DOI:** 10.1007/s00405-025-09816-9

**Published:** 2025-11-16

**Authors:** Fida Almuhawas, Dalal Alrushaydan, Shaza Saleh, Mada Aljabr, Hassan Yalcouy, Farid Alzahrani, Abdulrahman Alsanousi, Abdulrahman Hagr

**Affiliations:** 1 Department of Otolaryngology–Head and Neck Surgery, College of Medicine, King Saud University, Riyadh, Saudi Arabia; 2 Cochlear Arabia Regional Head Quarter, Riyadh, Saudi Arabia; 3 King Abdullah Ear Specialist Center (KAESC), King Saud University Medical City, Riyadh, Saudi Arabia

**Keywords:** Cochlear implants, Early activation, Switch on, Perimodiolar, Impedance, Sensorineural/surgery

## Abstract

**Background:**

Cochlear implantation (CI) is a proven treatment for severe-to-profound hearing loss, yet outcomes vary widely due to individual, device, and surgical factors. Intracochlear changes, such as fibrosis and neo-ossification, influence electrode impedance and device performance, potentially affecting speech perception.

**Purpose:**

This study investigates the effects of early activation (EA) versus standard activation on intracochlear impedance telemetry across different electrode types (lateral vs. perimodiolar). It aims to assess whether EA reduces the impedance and improves CI outcomes.

**Methods:**

This study involved 38 participants (54 CI ears) with Cochlear slim-straight or slim perimodiolar electrodes. Impedance telemetry was measured at four key time points, ranging from intra-operative to 12 months post-activation. Mixed-effects regression models evaluated the influence of activation timing and electrode type on impedance levels, with statistical significance determined using Bonferroni-corrected thresholds.

**Results:**

Perimodiolar electrodes demonstrated significantly lower impedances compared to lateral electrodes, particularly in EA cases. EA consistently resulted in reduced impedances across all cochlear regions, with the greatest reductions observed in the apical and middle regions at 3–6 months (*p* < 0.0001). By ≥ 1 year, impedance differences between activation modes diminished but remained significant in select regions (*p* = 0.012).

**Conclusion:**

EA, particularly with perimodiolar electrodes, optimizes the electrode-tissue interface, reducing impedance and potentially improving CI performance. These findings highlight the clinical benefits of EA in enhancing CI outcomes and support its consideration in routine CI protocols.

**Supplementary Information:**

The online version contains supplementary material available at 10.1007/s00405-025-09816-9.

## Introduction

Cochlear implantation (CI) has been established as a safe and effective treatment for individuals with severe to profound sensorineural hearing loss who derive limited benefit from conventional hearing aids [1]; Dazert* et al., [2]. Over the decades, CI technology has witnessed significant advancements, especially in electrode design, speech processing strategies, and connectivity, solidifying its status as a highly successful neural prosthesis (Athanasopoulos, Samara and Athanasopoulos [3].

Despite its clinical success, earlier studies mention variations in the outcomes among CI recipients, especially regarding speech perception, which ranges from little or no open-set speech understanding to robust comprehension even in challenging acoustic environments (Peterson, Pisoni and Miyamoto [4]. This variability can be attributed to a combination of individual factors, such as age, etiology, and cognitive abilities [5], as well as device-related and surgical factors, including electrode placement and insertion techniques [6–8]. Among these factors, intracochlear responses, such as fibrosis and neo-ossification, are emerging as critical contributors to variability in CI outcomes [9, 10].

Kamakura and Nadol [9], identified a relationship between poorer word recognition and the percent volume of neo-ossification within critical cochlear structures, including the scala tympani and scala media/vestibuli [9]. Moreover, insertion trauma, particularly to the basilar membrane, was found to be positively associated with the extent of neo-ossification [9]. These pathological changes not only impair speech perception they may also complicate future interventions, such as device reimplantation or regenerative therapies targeting neural or hair cell restoration (Foggia, Quevedo and Hansen [11].

Impedance telemetry, a vital tool for assessing electrode functionality and surrounding tissue conditions, offers valuable insights into these intracochlear changes [12]. It measures the electrical resistance across electrodes, providing information on short or open circuits and potential tissue changes adjacent to the electrode array (Asal, Sobhy and Massad [13]; Kumari et al., [14]. By leveraging telemetry data, clinicians can assess the degree of fibrosis or neo-ossification and its impact on CI performance, enabling more precise interventions [15]. Additionally, telemetry can be useful in monitoring and evaluating the factors that influence the development of fibrosis and neo-ossification post-CI in vivo, providing critical information for optimizing outcomes (Foggia, Quevedo and Hansen [11].

Routine activation of CIs typically occurs two to four weeks post-surgery to allow for tissue stabilization and healing [16]. However, emerging evidence suggests that early activation (EA), within a few days of implantation, is a safe and effective alternative [17]; Coelho, Shiao and Li [18]; Alahmadi et al., [19]; Yousef et al., 2025). Studies have demonstrated that early fitting accelerates the time required to achieve stable device programming, offers economic benefits, and facilitates quicker patient adaptation to the implant [20–22]; Sunwoo, Jeon and Choi [23]. Despite these advantages, the impact of EA on intracochlear fibrosis and its associated electrical impedances has not been fully explored. Fibrosis, which may alter the perilymph composition surrounding the electrodes, is thought to contribute to elevated impedances, further complicating device performance [24]; Duan, Clark and Cowan [25].

The present study evaluates the effects of early (next-day) versus standard (four-week post-surgery) activation on impedance telemetry measurements and examines the influence of electrode array type (lateral versus perimodiolar). These findings could provide insight into the impact of early electrical stimulation on the intracochlear foreign body response and/or insertion trauma following CIs, which may lead to the formation of fibrous tissue or neo-ossification. Additionally, the study explores whether the electrode type influences these outcomes.

## Methods

### Study design

This prospective study enrolled 38 patients from a tertiary cochlear implant (CI) center. A total of 55 CIs were initially included; however, one case lacked follow-up data and was excluded, yielding a final sample of 54 implanted ears. Of these, 30 involved lateral wall (LW) electrode arrays, while 24 involved perimodiolar arrays. The cohort comprised 21 unilaterally implanted and 17 bilaterally implanted patients, corresponding to 54 CI procedures. The gender distribution was approximately balanced, with 46.3% (*n* = 25) male and 53.7% (*n* = 29) female participants.

The mean age of the participants was 9.2 years (SD = 12.4), with age range from 0.9 to 63.7 years. Across the groups, EA was performed in 55.6% (*N* = 30) of cases, while late activation was conducted in 44.4% (*N* = 24).

#### Inclusion criteria

Recipients who were unilaterally or bilaterally implanted with Cochlear slim-straight electrodes (522 or 622) or PMelectrodes (532 or 632), provided written informed consent, and demonstrated a willingness to attend all study sessions.

#### Exclusion criteria

Exclusion criteria included the presence of cochlear malformations, a history of revision CI surgery, and cases of labyrinthitis ossificans secondary to meningitis or cochlear fracture. Additionally, patients with irregular clinic attendance, incomplete follow-up data, or follow-up disruptions due to the COVID-19 pandemic were excluded from the study.

## Procedure

Impedance telemetry, a measure of resistance to electrical current flow from the implant electrode to the spiral ganglion cells, was used to evaluate electrode functionality and the intracochlear environment. Impedance measurements provide insights into electrode integrity, compliance of the electrode map, and tissue conditions surrounding the electrode array. Early activation in this study was defined as fitting the external sound processor within 1–2 days following surgery, in line with protocols that have been in routine clinical use for over 15 years and have demonstrated safety and feasibility in previous studies** (**Alshalan et al., [19, 26]**).** Impedance measurements were performed at four key time points: (1) intra-operatively, (2) on the day of first activation, (3) at three to six months post-activation, and (4) 12 months post-activation.

## Data collection

Impedance telemetry was conducted using standardized clinical software, Custom Sound EP (version 6) and Custom Sound Suite (version 7.0). Data collection employed a predefined biphasic pulse configuration with a current level (CL) of 80 (equivalent to 74.2 µA), a pulse width (PW) of 25 µs, and an interphase gap (IPG) of 7 µs. Measurements were taken in multiple coupling modes, including common ground (CG) and monopolar configurations. The CG mode, where current flows between the active electrode and all other electrodes functioning as a single reference, was selected for analysis due to its broad representation of electrode performance and tissue interaction.

## Data management and statistical analysis

The primary endpoint for the analysis was the time point at three to six months post-activation, while the secondary endpoint was the time point ≤ 1-month post-activation. Common ground (CG) impedance data were used as observed criteria for the statistical models due to their normal distribution. Multiple regression analyses were conducted, with activation mode (early versus standard) and electrode type (LW PM) as fixed factors. Mixed-effects models were utilized to examine the trajectories of CG impedance levels over time, treating patients as a random block effect to account for within-patient variation.

To control the comparison-wise error rate, the Bonferroni correction was applied, dividing the observed p-values by a factor of k = 3. Statistical analyses were conducted using SAS software version 9.4 (SAS Institute, Cary, NC, USA). Results were deemed statistically significant if the adjusted p-value was < 0.05.

## Ethical considerations

The study protocol was reviewed and approved by the appropriate ethics committee (IRB approval number E-21–6316). Written informed consent was obtained from all participants or their guardians prior to the initiation of study-related procedures.

## Results

The present study evaluated the influence of electrode type and activation mode on intracochlear impedance telemetry in 54 CI ears. The sample comprised 30 cases with lateral electrodes and 24 cases with perimodiolar electrodes. Activation mode was evenly distributed, with 30 cases undergoing EA and 24 receiving standard activation. Statistical analysis confirmed the balance between study groups, as indicated by the homogeneity test (*p*-value = 0.71).

### Demographic analysis

#### Age

The demographic analysis highlighted variations in age across the study groups defined by activation mode and electrode type. Among the 54 CI cases, the mean age was 9.2 years (SD = 12.4), with a median age of 3.4 years and a broad age range from 0.9 to 63.7 years, reflecting the diversity of the cohort. In the EA group, the mean age was 12.1 years (SD = 15.12), with lateral electrode recipients averaging 13.5 years and PM electrode recipients averaging 10.5 years.

In the late activation group, the mean age was 5.5 years (SD = 6.41), with lateral electrode recipients having a mean age of 4.5 years and PM recipients averaging 7.0 years. LW recipients exhibited a broader age range (1.4 to 63.7 years) compared to perimodiolar recipients (0.9 to 29.2 years) (Table 1). These findings illustrate the demographic variability within the study population, which may influence the generalizability and outcomes of the analysis.

**Table 1:** Demographic analysis

Mode of activation			
Electrode type	Early	Late	All
Lateral	16 (53.3%)	14 (46.7%)	30
Perimodiolar	14 (58.3%)	10 (41.7%)	24
Total	30 (55.6%)	24 (44.4%)	54
Chi2-statistic: 0.14, df = 1, p-value = 0.713 Note: %-fractions in the inner cells are calculated by row.			
Distribution of gender by study groups			
	Gender		
Electrode/Activation	Male	Female	Total
Lateral/Early	13 (43.3%)	17 (56.7%)	30
Perimodiolar/Late	12 (50.0%)	12 (50.0%)	24
Total	25 (46.3%)	29 (53.7%)	54
Chi2-statistic: 0.24, df = 1, p-value = 0.625 Note: %-fractions in the inner cells are calculated by row			
	Etiology		
Study group	Congenital	Progressive	Total
Lateral-Early	12 (75.0%)	4 (25.0%)	16
Lateral-Late	13 (92.9%)	1 (7.1%)	14
Perimodiolar-Early	9 (64.3%)	5 (35.7%)	14
Perimodiolar-Late	9 (90.0%)	1 (10.0%)	10

#### Gender

The analysis of gender distribution across study groups indicates a balanced representation between male and female participants. Among the 54 cases, 46.3% (*n* = 25) were male and 53.7% (*n* = 29) were female. For lateral electrode recipients, 43.3% (*n* = 13) were male, while 56.7% (*n* = 17) were female. For perimodiolar electrode recipients, an equal distribution was observed, with 50% (*n* = 12) male and 50% (*n* = 12) female participants (Table 1).

In terms of activation mode, EA cases included 43.3% (*n* = 13) males and 56.7% (*n* = 17) females, whereas late activation cases comprised an even split of 50% (*n* = 12) males and 50% (*n* = 12) females (Table 1). The chi-square test results (χ² = 0.24, *p* = 0.625) confirm that there were no statistically significant differences in gender distribution across the electrode types or activation modes, highlighting an equitable gender composition across the study groups.

#### Etiology

Congenital hearing loss was the predominant etiology across all groups, accounting for 79.6% (*n* = 43) of cases, while progressive hearing loss represented 20.4% (*n* = 11). Among lateral electrode recipients, congenital hearing loss was observed in 75% (*n* = 12) of the EA group and 92.9% (*n* = 13) of the late activation group. Similarly, for PM electrodes, congenital hearing loss was present in 64.3% (*n* = 9) of the EA group and 90% (*n* = 9) of the late activation group (Table 1). 

### Effect of mode of activation on CG impedances

**Fig. 1:**
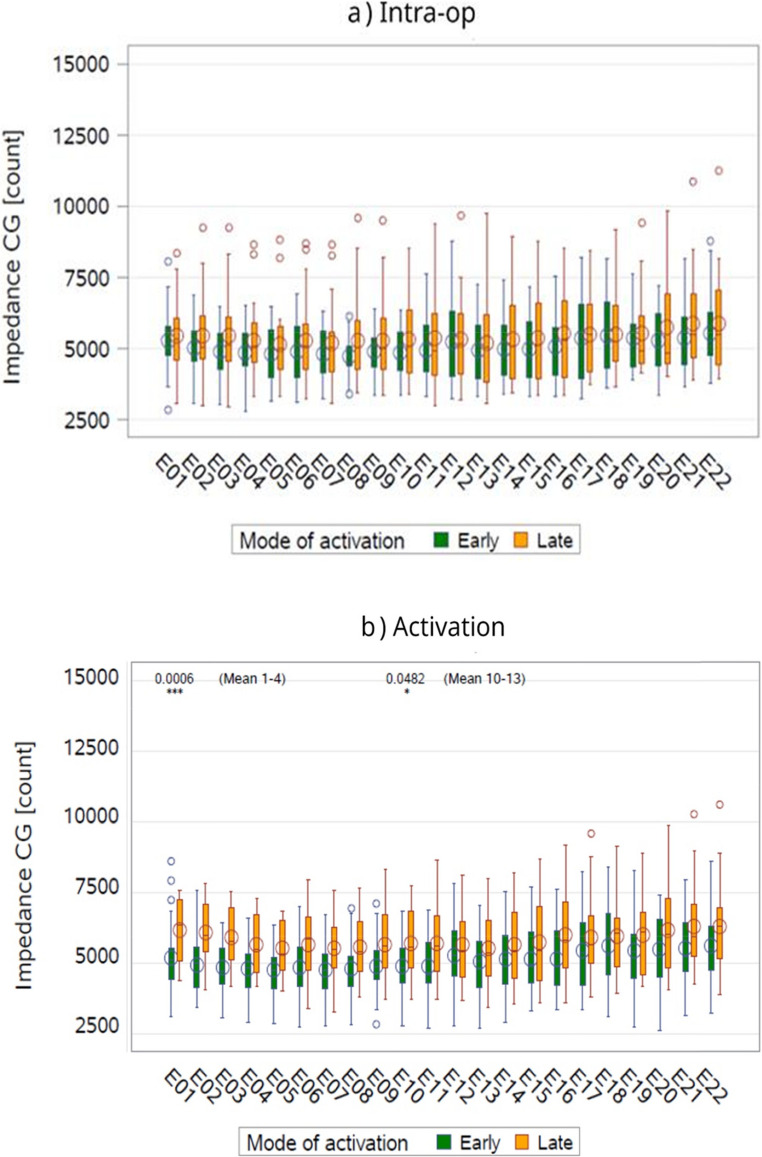

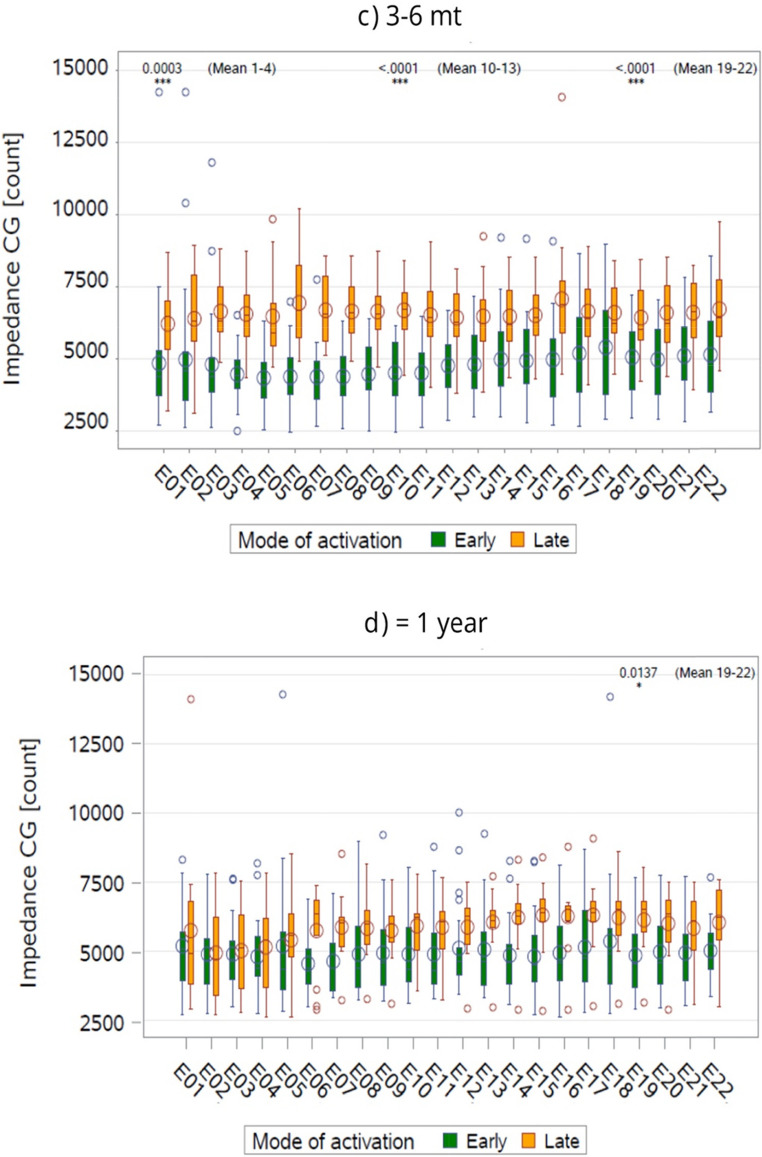
Boxplots of CG count data of electrodes by activation mode and by time-point. This figure presents boxplots comparing CG impedance values between EA and late activation groups across four time points: intra-operative, activation, 3–6 months, and ≥1 year. Lower impedance values were consistently observed in the EA group, with statistically significant differences emerging at activation and 3–6 months, particularly in the basal and apical regions. The plots illustrate central tendency and variability in impedance data over time. CG= common ground; Intra-op= intra operative

Impedance telemetry results revealed notable differences in CG impedances between early and late activation groups across time points and cochlear regions. EA was consistently associated with lower impedances compared to late activation, as observed in boxplots and confirmed through regression analyses. The effect of mode of activation was examined with more precision by means of the regression models, shown in Fig. 1.

In the basal region, EA demonstrated significantly lower mean impedances than late activation at activation (4932.7 vs. 5921.4, p = 0.0007) and 3–6 months (4826.1 vs. 6463.1, p = 0.0003). However, at ≥1 year, no statistically significant difference was observed (4776.0 vs. 5221.9, p = 0.3441). During intra-op, EA showed lower mean impedances compared to late activation (5005.6 vs. 5405.5, p = 0.1829), though this difference was not statistically significant (Table 2).

For the middle region, EA was associated with lower impedances at all time points. The differences were statistically significant at activation (5002.4 vs. 5517.8, *p* = 0.0635), 3–6 months (4615.3 vs. 6507.8, *p* < 0.0001), and ≥ 1 year (4769.0 vs. 5953.0, *p* = 0.0137). The most pronounced difference occurred at 3–6 months. During intra-op, EA also exhibited lower mean impedances than late activation (4951.2 vs. 5182.4, *p* = 0.4727), though the difference was not statistically significant (Table 2).

In the apical region, EA also resulted in lower impedances compared to late activation. The differences were marginally significant at activation (5476.9 vs. 6044.5, *p* = 0.0751) but became highly significant at 3–6 months (5004.5 vs. 6540.7, *p* < 0.0001) and ≥ 1 year (4916.1 vs. 6044.4, *p* = 0.0120). Intra-op results showed lower mean impedances for EA compared to late activation (5342.5 vs. 5611.7, *p* = 0.3875), though this difference was not statistically significant (Supplementary Table 2). 

**Table 2:** Lsmeans and 95% CI by time point and location for activation mode

Time Point	Location	Type	LSMEAN	Standard Error	Lower CL	Upper CL	Pr > |t|
Intra-op	Basal (Mean 1-4)	Early	5005.6	205.1	4593.9	5417.3	0.1829
		Late	5405.5	215.3	4973.2	5837.8	_
	Middle (Mean 10-13)	Early	4951.2	221.3	4506.9	5395.5	0.4727
		Late	5182.4	232.4	4715.9	5648.8	_
	Apical (Mean 19-22)	Early	5342.5	213.9	4913.1	5771.9	0.3875
		Late	5611.7	224.6	5160.9	6062.6	_
Activation	Basal (Mean 1-4)	Early	4932.7	183.9	4562.9	5302.5	0.0007
		Late	5921.4	204.9	5509.5	6333.3	_
	Middle (Mean 10-13)	Early	5002.4	182.0	4636.5	5368.3	0.0635
		Late	5517.8	202.7	5110.3	5925.3	_
	Apical (Mean 19-22)	Early	5476.9	209.2	5056.2	5897.5	0.0751
		Late	6044.5	233.0	5576.0	6513.0	_
3 - 6 months	Basal (Mean 1-4)	Early	4826.1	284.5	4252.7	5399.5	0.0003
		Late	6463.1	302.7	5853.0	7073.2	_
	Middle (Mean 10-13)	Early	4615.3	205.6	4200.9	5029.7	<.0001
		Late	6507.8	218.8	6066.9	6948.7	_
	Apical (Mean 19-22)	Early	5004.5	222.5	4556.1	5452.9	<.0001
		Late	6540.7	236.7	6063.6	7017.8	_
>= 1 year	Basal (Mean 1-4)	Early	4776.0	313.6	4127.2	5424.7	0.3441
		Late	5221.9	338.7	4521.2	5922.6	_
	Middle (Mean 10-13)	Early	4769.0	297.8	4157.9	5380.0	0.0137
		Late	5953.0	338.0	5259.5	6646.4	_
	Apical (Mean 19-22)	Early	4916.1	278.0	4345.6	5486.5	0.0120
		Late	6044.4	315.5	5397.0	6691.8	_

**Fig. 2:**
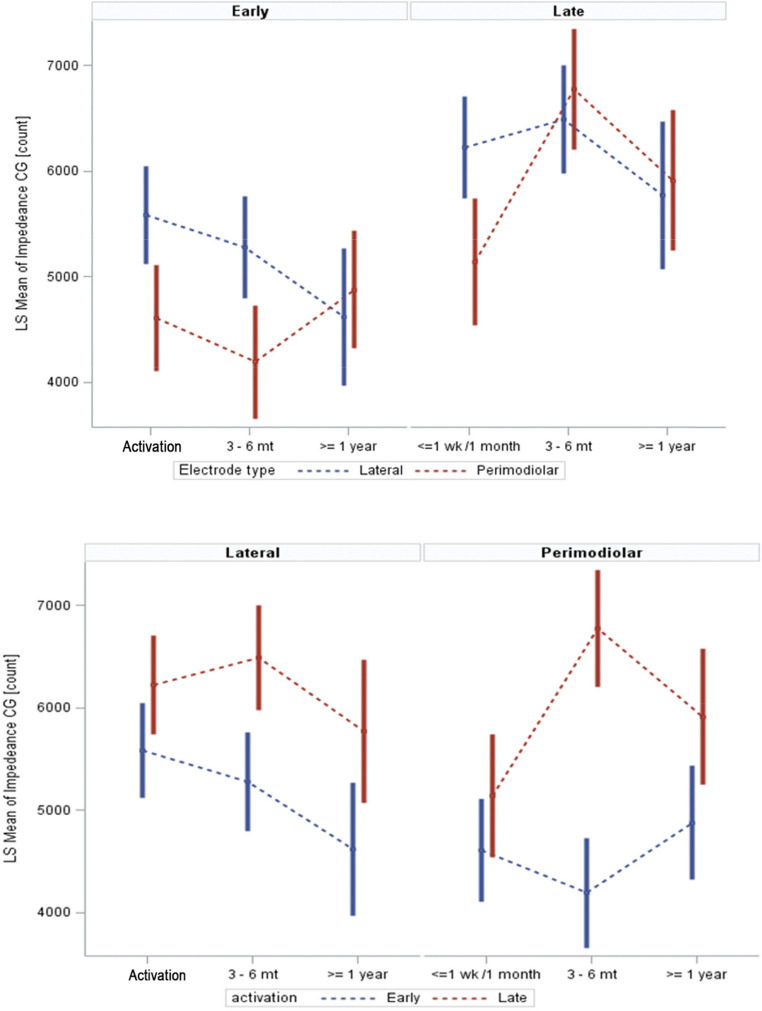
Time-Dependent Impedance Trends by Activation Mode and Electrode Location. This line graph shows longitudinal trends in CG impedance for both early and late activation groups across different cochlear regions, basal, middle, and apical. Each line represents the mean impedance trajectory over time, demonstrating the temporal evolution of electrode-tissue interaction. EA is associated with lower and more stable impedance profiles, particularly in the middle and apical regions, suggesting reduced inflammatory or fibrotic response. CG = common ground; LS = Least Squares

**Fig. 3:**
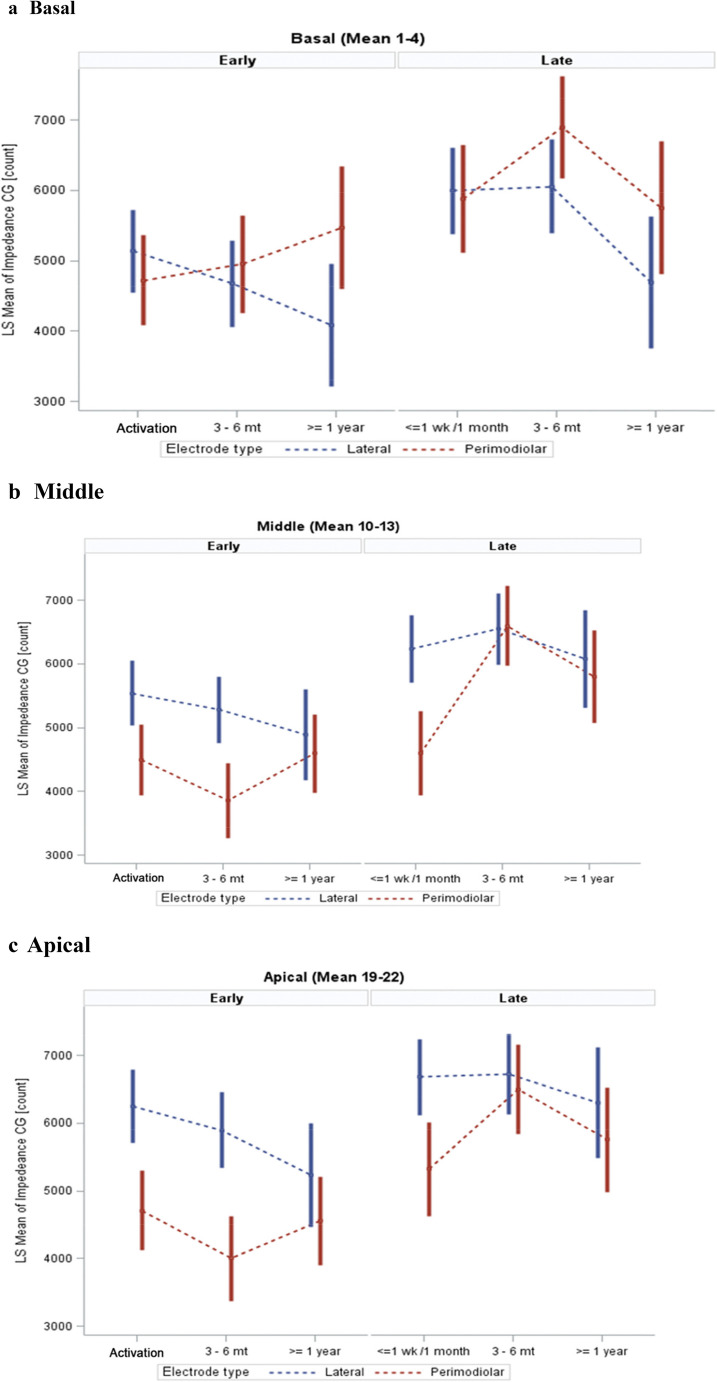
Time-Dependent Impedance Trends Across Cochlear Regions. This figure illustrates CG impedance trends over time (intra-op to ≥ 1 year) stratified by cochlear region, basal, middle, and apical. The curves highlight that the greatest reductions in impedance over time occurred in the apical and middle regions, supporting the hypothesis of region-specific tissue responses to electrical stimulation. These findings emphasize the anatomical influence on post-implant impedance changes. CG = common ground; LS = Least Squares

The analysis revealed significant interactions between activation mode, time, and cochlear region. EA demonstrated a distinct pattern of reducing impedances over time, particularly in the middle and apical regions. These findings indicate that EA may attenuate impedance increases commonly associated with CI, potentially improving device performance over time (Figs. 2 and 3).

###  Effect of electrode array type on CG impedances

**Fig. 4:**
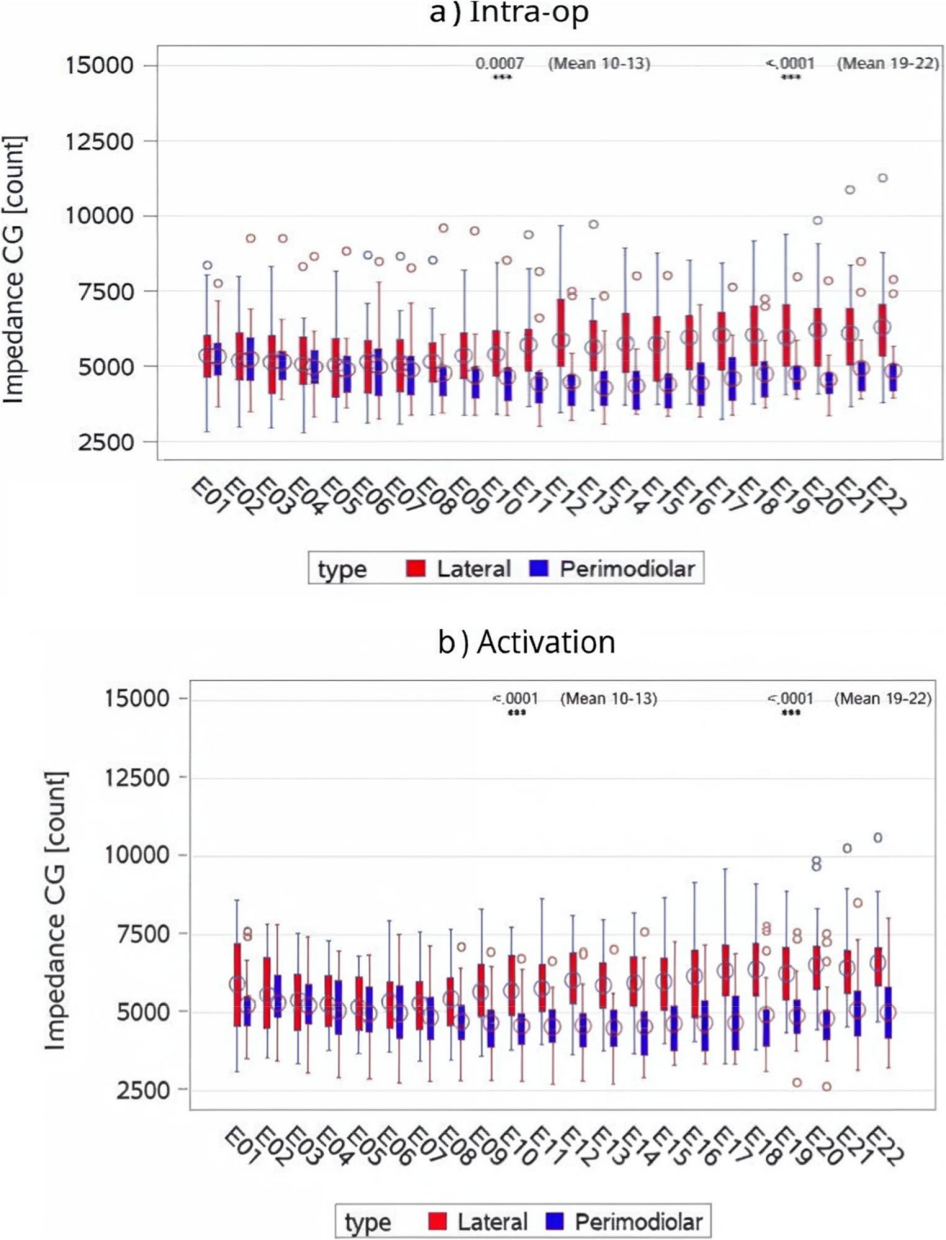

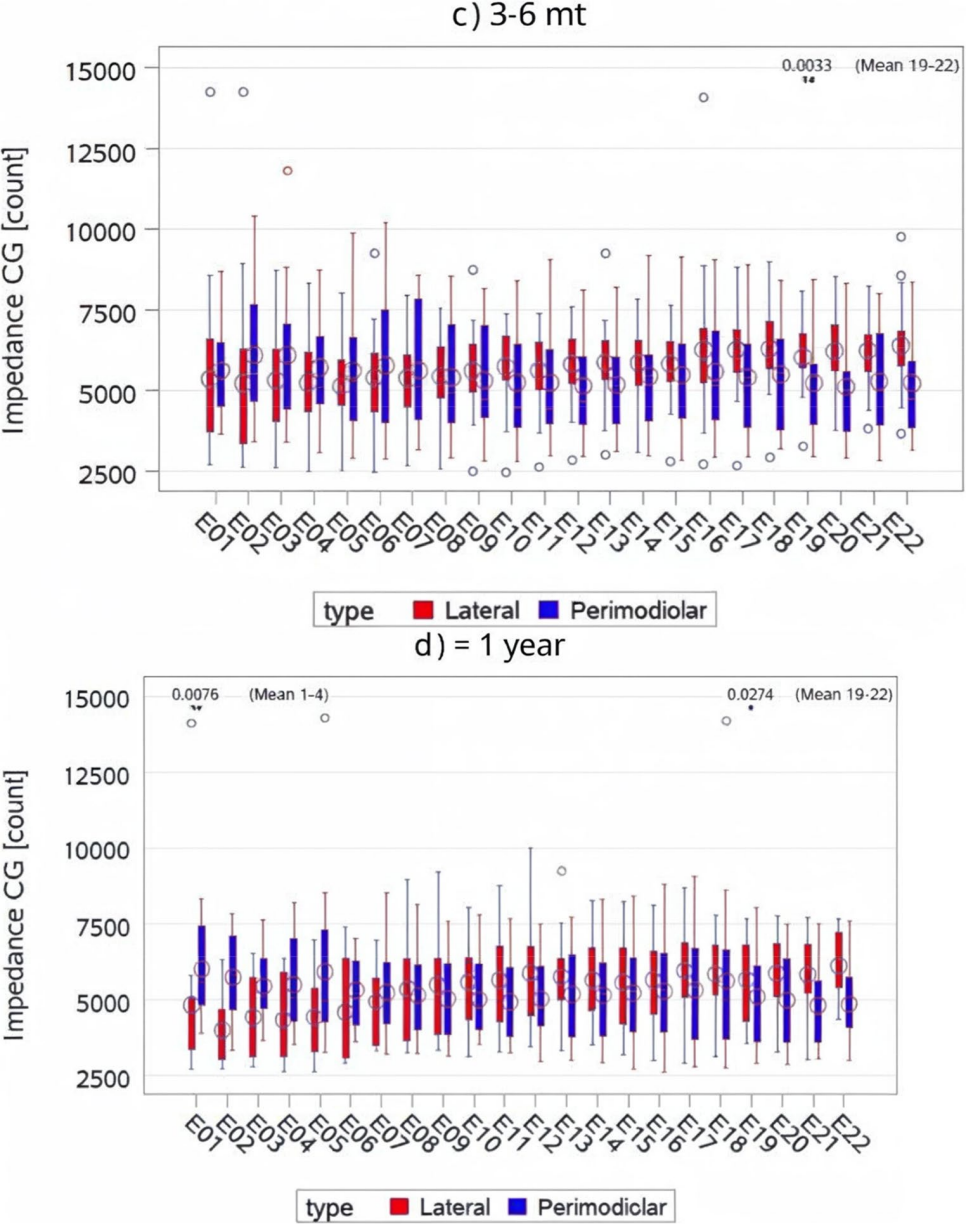
Electrode Impedance (CG Count) by Electrode Type and Time Point. This figure illustrates mean CG impedance values across time points (intra-operative, activation, 3–6 months, and ≥ 1 year) for both PM and LW electrodes. Impedance values are shown separately for apical, middle, and basal regions of the cochlea. Perimodiolar electrodes generally exhibited lower impedances over time, particularly in the middle and apical regions. CG = common ground; LS = Least Squares

PM devices consistently demonstrated lower impedances compared to LW devices, particularly in the middle and most apical regions. In the middle region (Fig. 4), PM electrodes demonstrated significantly lower mean impedances compared to LW electrodes at intra-op (*p* = 0.0007) and activation (*p* < 0.0001), with the disparity reducing over time, becoming marginally significant at 3–6 months (*p* = 0.0518) and nonsignificant at ≥ 1 year (*p* = 0.4696).

In the apical region (Fig. 4), it was noted that perimodiolar electrodes consistently displayed significantly lower mean impedances than LW electrodes at activation (*p* < 0.0001), 3–6 months (*p* = 0.0033), and ≥ 1 year, except at intra-op, where the trend reversed without reaching statistical significance (*p* = 0.9748).

In the basal region, it was observed that mean impedances for perimodiolar electrodes were slightly higher than LW electrodes at intra-op (*p* = 0.9748) and marginally lower at activation (*p* = 0.3149) and 3–6 months (*p* = 0.1978), though these differences were not statistically significant. These observations highlight region- and time-dependent variations in impedance, suggesting distinct interactions between electrode types and the surrounding cochlear tissues (Fig. 4).

## Discussion

The findings of the present study indicate that PM electrodes, when combined with EA of CIs, are associated with consistently lower electrode impedance values compared to LW electrodes and late activation. This highlights the potential role of EA, particularly in conjunction with perimodiolar electrodes, in optimizing the intracochlear environment and enhancing device performance. This effect was observed across multiple time points and cochlear regions, underscoring the potential benefits of early electrical stimulation in modulating the cochlear environment post-implantation. The impact of EA was particularly evident in the apical and middle cochlear regions, where significant reductions in impedance were noted, suggesting a region-specific influence of EA on the intracochlear tissue response.

Lower electrode impedance values are advantageous for several reasons. Impedance is directly related to the electrical resistance at the electrode-tissue interface, and high impedances necessitate higher voltage outputs to achieve adequate stimulation currents (Sunwoo, Jeon and Choi [23]; Tejani et al., [27]. This can lead to rapid battery depletion and increased current spread, potentially reducing the precision of neural stimulation and impairing frequency resolution [7, 28, 29]. Numerous studies have linked higher impedances with suboptimal auditory performance, emphasizing the importance of maintaining low impedance levels for effective CI functioning [30, 31].

In this study, EA appears to mitigate the rise in impedance commonly associated with fibrosis and inflammatory responses. By lowering impedance values, EA may enhance device longevity and efficiency, particularly in energy-demanding scenarios such as thick skin flaps or complex cochlear anatomies [19]. Previous studies, such as Velandia et al., [32], reported lower impedances in perimodiolar arrays compared to LW electrodes, aligning with findings of the present study.

However, this study expands upon prior work by showing that the impedance-lowering effect of perimodiolar arrays was pronounced only with EAs, suggesting an interaction between electrode type and activation timing. Specifically, perimodiolar electrodes demonstrated significantly lower impedances compared to lateral electrodes in EA patients, however this advantage was not observed in late-activated groups. These findings suggest that the benefits of perimodiolar arrays may be contingent upon early activation, potentially due to their closer proximity to neural structures and reduced current spread.

Several biological mechanisms may explain the observed benefits of EA on electrode impedance. Early electrical stimulation may modulate the immune response by reducing the inflammatory cascade and fibrosis formation around the electrode array [33]. Electrical stimulation has been shown to alter protein adsorption, decrease inflammatory cell proliferation, and activate anti-inflammatory pathways, thereby maintaining a healthier electrode-tissue interface [24, 33]. Additionally, EA may prevent the degeneration of spiral ganglion neurons (SGN), which are critical for effective auditory signaling [33].

The present study revealed region-specific effects of early, with the most pronounced reductions in the apical and middle cochlear regions. This pattern could reflect anatomical and physiological differences along the cochlear spiral (Leone, Mosca and Grassia [34]. The apical region, which processes low-frequency sounds, may be more susceptible to inflammatory and fibrotic reactions due to its narrower ductal dimensions and closer proximity of electrodes to the medial cochlear wall [35]. Early stimulation might counteract these responses more effectively in the apical region, reducing impedance values and preserving residual hearing in this critical area.

The findings align with previous studies that report lower impedances in perimodiolar arrays, however this study uniquely highlights the interaction between EA and electrode type. Earlier research, such as [36], found no significant long-term differences in impedance values between activation groups, however this may be attributed to differences in follow-up duration, sample size, or electrode design. The sustained reductions in impedance observed in this study underline the potential long-term benefits of EA, which warrants further investigation.

This study adds to existing knowledge by examining activation timing and electrode array type in combination, rather than as separate factors, in relation to impedance telemetry outcomes. The findings suggest that the lower impedance observed with PM electrodes may be more evident when EA is applied, indicating a possible interaction between surgical and programming variables. Furthermore, the region-specific analysis of impedance across the basal, middle, and apical cochlear segments provides additional context for understanding spatial variation in electrode-tissue responses. The use of a prospective, cross-sectional design with follow-up extending to 12 months post-activation allows for the observation of both early and longer-term impedance trends. These observations may contribute to the ongoing refinement of clinical decision-making in cochlear implant care.

The study provides valuable insights into the effects of EA and electrode type on impedance; however, certain limitations should be acknowledged. The sample size, although sufficient for the primary analysis, may not fully capture the variability present in diverse CI populations. Furthermore, while the prospective design facilitated greater control over variables such as surgical techniques and follow-up care, variability in patient adherence and other unmeasured factors may still have influenced the results. One notable limitation of this study is the age distribution of the sample, which was heavily skewed toward pediatric patients, with a mean age of 9.2 years and a median age of 3.4 years. While a wide age range was represented, including patients up to 63.7 years, the findings may not fully generalize to adult CI recipients, who may exhibit different patterns of tissue healing, immune response, or electrode-tissue interface properties. Future studies with age-stratified cohorts are warranted to validate these findings in older populations.

Future research should focus on the long-term auditory and quality-of-life outcomes associated with EA, as well as the underlying biological mechanisms driving the observed effects. Further analyses with trans impedance telemetry might shed more light on that effect on fibrosis formation particularly in terms of in vivo investigations.

## Conclusion

This study highlights that PM electrodes exhibit lower CG impedances compared to LW electrodes, primarily in EA patients, with this difference diminishing in late-activated groups after the first month. Early activation demonstrated reduced CG impedances across all electrode types and regions, highlighting its role in optimizing the electrode-tissue interface and mitigating inflammatory responses.

## Supplementary Information

Below is the link to the electronic supplementary material.

Supplementary Material 1 (DOCX. 16.9 KB)
