# Supplementary material for: Effect of early activation and perimodiolar electrodes on cochlear implant impedance
Source: Eur Arch Otorhinolaryngol. 2025 Nov 16;283(3):1597–609. doi: 10.1007/s00405-025-09816-9 (PMC13002729; doi:10.1007/s00405-025-09816-9)
Supplement: Supplementary file 1 — Supplementary Material 1 (DOCX. 16.9 KB) [file 405_2025_9816_MOESM1_ESM.docx]

**Supplementary Information**

Supplementary Table 1: Lsmeans and 95% CI by time point and location for activation mode.

| Time Point | Location | Type | LSMEAN | Standard Error | Lower CL | Upper CL | Pr > \|t\| |
| --- | --- | --- | --- | --- | --- | --- | --- |
| Intra-op | Basal (Mean 1–4) | Early | 5005.6 | 205.1 | 4593.9 | 5417.3 | 0.1829 |
|  |  | Late | 5405.5 | 215.3 | 4973.2 | 5837.8 |  |
|  | Middle (Mean 10–13) | Early | 4951.2 | 221.3 | 4506.9 | 5395.5 | 0.4727 |
|  |  | Late | 5182.4 | 232.4 | 4715.9 | 5648.8 |  |
|  | Apical (Mean 19–22) | Early | 5342.5 | 213.9 | 4913.1 | 5771.9 | 0.3875 |
|  |  | Late | 5611.7 | 224.6 | 5160.9 | 6062.6 |  |
| Activation | Basal (Mean 1–4) | Early | 4932.7 | 183.9 | 4562.9 | 5302.5 | **0.0007** |
|  |  | Late | 5921.4 | 204.9 | 5509.5 | 6333.3 |  |
|  | Middle (Mean 10–13) | Early | 5002.4 | 182.0 | 4636.5 | 5368.3 | 0.0635 |
|  |  | Late | 5517.8 | 202.7 | 5110.3 | 5925.3 |  |
|  | Apical (Mean 19–22) | Early | 5476.9 | 209.2 | 5056.2 | 5897.5 | 0.0751 |
|  |  | Late | 6044.5 | 233.0 | 5576.0 | 6513.0 |  |
| 3–6 months | Basal (Mean 1–4) | Early | 4826.1 | 284.5 | 4252.7 | 5399.5 | **0.0003** |
|  |  | Late | 6463.1 | 302.7 | 5853.0 | 7073.2 |  |
|  | Middle (Mean 10–13) | Early | 4615.3 | 205.6 | 4200.9 | 5029.7 | **< .0001** |
|  |  | Late | 6507.8 | 218.8 | 6066.9 | 6948.7 |  |
|  | Apical (Mean 19–22) | Early | 5004.5 | 222.5 | 4556.1 | 5452.9 | **< .0001** |
|  |  | Late | 6540.7 | 236.7 | 6063.6 | 7017.8 |  |
| >= 1 year | Basal (Mean 1–4) | Early | 4776.0 | 313.6 | 4127.2 | 5424.7 | 0.3441 |
|  |  | Late | 5221.9 | 338.7 | 4521.2 | 5922.6 |  |
|  | Middle (Mean 10–13) | Early | 4769.0 | 297.8 | 4157.9 | 5380.0 | 0.0137 |
|  |  | Late | 5953.0 | 338.0 | 5259.5 | 6646.4 |  |
|  | Apical (Mean 19–22) | Early | 4916.1 | 278.0 | 4345.6 | 5486.5 | **0.0120** |
|  |  | Late | 6044.4 | 315.5 | 5397.0 | 6691.8 |  |
